# Electron-donating functional groups strengthen ligand-induced chiral imprinting on CsPbBr_3_ quantum dots

**DOI:** 10.1038/s41598-023-50595-2

**Published:** 2024-01-03

**Authors:** Wiley A. Dunlap-Shohl, Nazifa Tabassum, Peng Zhang, Elizabeth Shiby, David N. Beratan, David H. Waldeck

**Affiliations:** 1 Department of Chemistry, University of Pittsburgh, Pittsburgh, 15213 USA; 2 Department of Chemistry, Duke University, Durham, 27708 USA; 3 Department of Physics, Duke University, Durham, 27705 USA; 4 Department of Biochemistry, Duke University, Durham, 27710 USA

**Keywords:** Quantum dots, Electronic materials

## Abstract

Chiral perovskite nanoparticles and films are promising for integration in emerging spintronic and optoelectronic technologies, yet few design rules exist to guide the development of chiral material properties. The chemical space of potential building blocks for these nanostructures is vast, and the mechanisms through which organic ligands can impart chirality to the inorganic perovskite lattice are not well understood. In this work, we investigate how the properties of chiral ammonium ligands, the most common organic ligand type used with perovskites, affect the circular dichroism of strongly quantum confined CsPbBr_3_ nanocrystals. We show that aromatic ammonium ligands with stronger electron-donating groups lead to higher-intensity circular dichroism associated with the lowest-energy excitonic transition of the perovskite nanocrystal. We argue that this behavior is best explained by a modulation of the exciton wavefunction overlap between the nanocrystal and the organic ligand, as the functional groups on the ligand can shift electron density toward the organic species-perovskite lattice interface to increase the imprinting.

## Introduction

Over the past decade, halide perovskites have demonstrated outstanding optoelectronic properties that make them attractive for photovoltaics^1–3^, LEDs^4,5^, detectors^6–8^, and light sources. Halide perovskite quantum dots (QDs) are particularly promising for light-emitting applications because of their high photoluminescence quantum yield (PLQY), narrow emission linewidths, and tunability from the near-infrared through the ultraviolet^9,10^. Perovskite nanocrystals have also shown promise as single-photon emitters^11,12^, and controlling the photon quantum state would make them potential candidates for quantum applications in computing and imaging. Among the many classes of nanomaterials, halide perovskite nanocrystals are most directly comparable to those made of other semiconducting compounds that possess band gaps in or near the visible range, such as chalcogenides (e.g., CdSe) or pnictides (e.g., InP). Unlike these more conventional semiconductors, however, the interatomic bonding interactions in halide perovskites are significantly more ionic than covalent, with the component ions having low valences ^13,14^. Perovskites thus possess a relatively soft crystal lattice, which introduces distinct advantages and disadvantages^13^. On one hand, halide perovskite nanocrystals form easily at low temperatures and may even be synthesized and compositionally modified under ambient conditions, creating a promising outlook for low-cost commercial technologies^13,14^. Moreover, although defects such as halide vacancies readily form in perovskites, the optoelectronic properties are robust to the presence of the most thermodynamically favorable defects, and perovskite nanocrystals do not require surface passivation to attain near-unity PLQYs (unlike chalcogenide nanocrystals), further simplifying synthesis of high-quality materials. The lead halide chemistry of perovskites is proposed to play a role in this defect tolerance in that the band edges are predominantly composed of antibonding orbitals, making the associated energy levels likely to lie near the edges of the band gap (or in the bands themselves) as opposed to deep within it^14,15^. The defect tolerance may also be partially attributable to the soft lattice, which has been proposed to facilitate the formation of polarons that screen Coulombic interactions between charge carriers and defects^14,16,17^. While the chemistry and ionicity of halide perovskite nanocrystals confer compelling advantages over conventional semiconductors, they are also less thermally and environmentally stable than their more covalent cousins and are vulnerable to degradation by the ambient environment over the long term, which remains an ongoing challenge for the halide perovskite field at large^18^. If these obstacles can be addressed, however, the commercial prospects for perovskite nanocrystal-based optoelectronic devices are very exciting.

Introducing chirality into perovskite nanostructures offers the potential to harness additional modes of optoelectronic functionality. The symmetry breaking that manifests from chiral imprinting onto the electronic states of perovskite nanoparticles^19,20^, and hybrid organic–inorganic perovskite films^21^, affords a degree of control over the angular momentum of both charge carriers and photons, thus opening the possibility of selecting the polarization of emitted and absorbed light. Via the chiral-induced spin-selectivity effect, electric currents passing through such materials will be filtered according to the carriers’ spin orientation^22–25^. Furthermore, chiral molecules can imprint circular dichroism (CD) onto electronic transitions of the inorganic frameworks to which they bind. Although these properties promise to make chiral perovskites useful in new classes of opto-spintronic devices that are responsive to circularly polarized light, the underlying effects are often weak^26^, making it important to understand how to enhance the chiral imprinting between the organic structure and the perovskite lattice.

Many organoammonium cations have been used to imprint chirality onto perovskite structures^27,28^, but details of the imprinting mechanism are poorly understood and there are few rules to guide the design of the chiral ligands. Recently, Tabassum et al.^29^ showed that reducing the size of the CsPbBr_3_ perovskite nanocrystals, thereby pushing their electronic structure further into the strong quantum confinement regime, has a dramatic effect on chiral imprinting. They found that the CD strength increases exponentially with the inverse of the particle diameter. Georgieva et al.^19,30^ found that different ligand chemistries affect chiral imprinted CD spectra of perovskite nanoplatelets, and Hubley et al.^31^ found a robust correlation between circularly polarized photoluminescence and the CD strength in similar systems. Here, we study how varying the functional group at the para position of the phenyl ring in chiral ligands, (*R*/*S*)-4-X-phenethylammonium, affects the circular dichroism strength for the lowest-energy exciton transition in strongly quantum confined (~ 2 nm diameter) CsPbBr_3_ perovskite nanocrystals. We explore the possible ways by which changes in the electron density, induced by the functional group of the chiral adsorbate at the ligand-nanoparticle interface, influence the induced CD strength. We find that the more strongly electron-donating is the functional group, the stronger is the induced CD amplitude. We consider several mechanistic hypotheses that may underpin this behavior and conclude that the observation is most likely due to increasing overlap between the frontier orbital wavefunctions of the ligand and the perovskite nanocrystal.

## Results and discussion

### Synthesis and optical properties of chiral quantum dots

**Figure 1:**
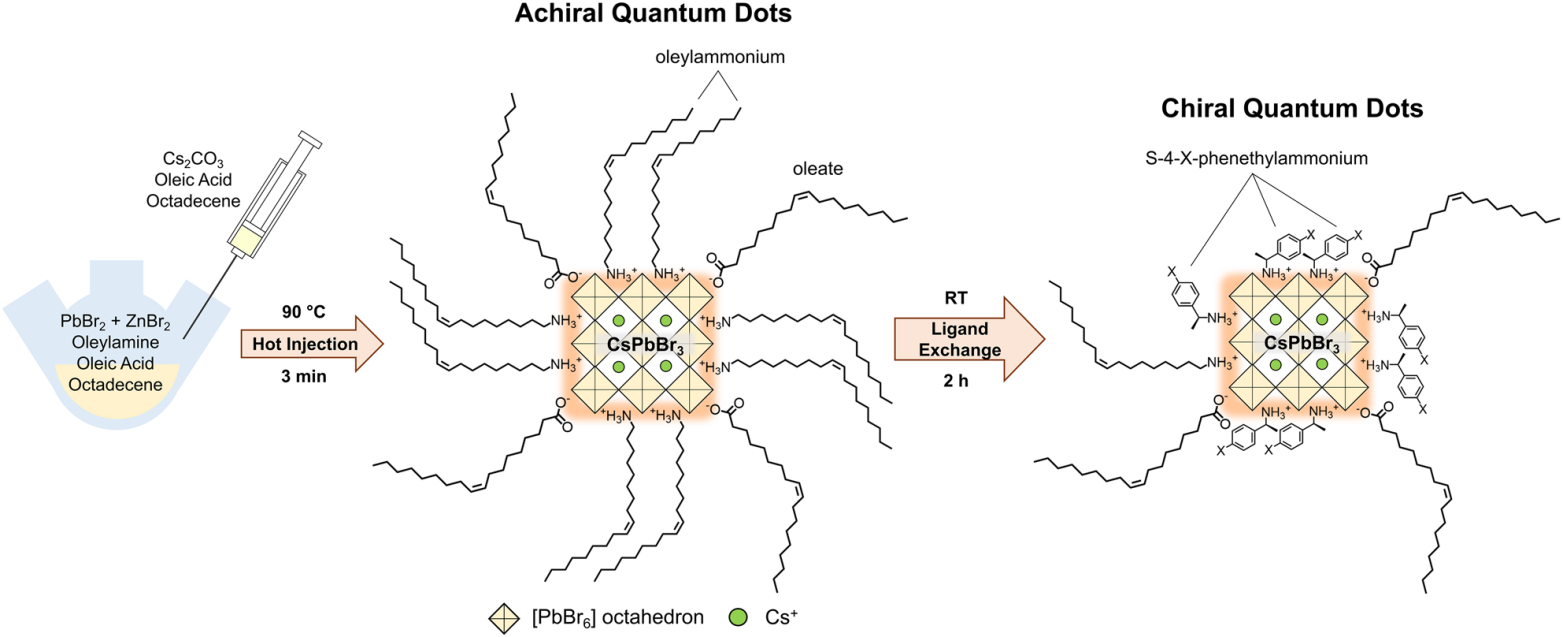
Synthesis procedure for forming chiral perovskite QDs. Achiral CsPbBr_3_ quantum dots are prepared by hot injection of Cs oleate into a solution of PbBr_2_, ZnBr_2_, oleylamine, and oleic acid in octadecene, yielding particles approximately 2 nm in diameter that are decorated with oleylamine and oleate ligands. As in our previous work^20,29^, ZnBr_2_ is used in large excess to increase the chemical potential of Br^−^ during synthesis, which helps to maintain a small particle diameter, as reported by Dong et al.^32^. To imprint chirality onto the particles, chiral *R*- or *S*-4-X-phenethylammonium bromide is introduced to the dispersions and incubated for 2 h, during which the chiral ligands mostly displace the native oleylammonium and oleate ligands.

The procedure for synthesizing the chiral perovskite quantum dots is summarized below and in Fig. 1; further details are given in the Methods section. Colloidal CsPbBr_3_ QDs of nominal size 2 nm were prepared by a conventional hot-injection method from PbBr_2_ and Cs oleate precursors, using oleylamine and oleic acid as stabilizing ligands, as we have previously detailed elsewhere^20,29^. ZnBr_2_ was added in approximately 12-fold excess to suppress nanocrystal growth and maintain strong quantum confinement^32^. Excess native ligands were then removed by centrifugation and the resulting pellet, comprising purified 2 nm CsPbBr_3_ QDs, was collected and redispersed in anhydrous toluene. UV–Vis spectra of the achiral particles after purification (of which a representative example is given in Fig. S1) exhibit a mean exciton peak position of 428.7 ± 0.5 nm (uncertainty represented by the standard deviation)**,** consistent with our previous reports wherein the exciton peak lies at 428 nm^20,29^. Chirality was imprinted upon the QDs by injecting tert-butanol solutions of (*R/S*)-4-X-phenethylammonium bromide ((*R/S*)-4-X-PEABr; X = CH_3_, H, F, Br) into the QD dispersions and incubating them at room temperature, during which period the chiral ligands partially displace the remaining native ligands from the QD surfaces (Fig. S2). The excess chiral ligands were then removed by centrifugation, and the resulting perovskite QD pellets were collected and redispersed in toluene for optical property characterization by UV–Vis-NIR absorbance and circular dichroism spectroscopy.

**Figure 2:**
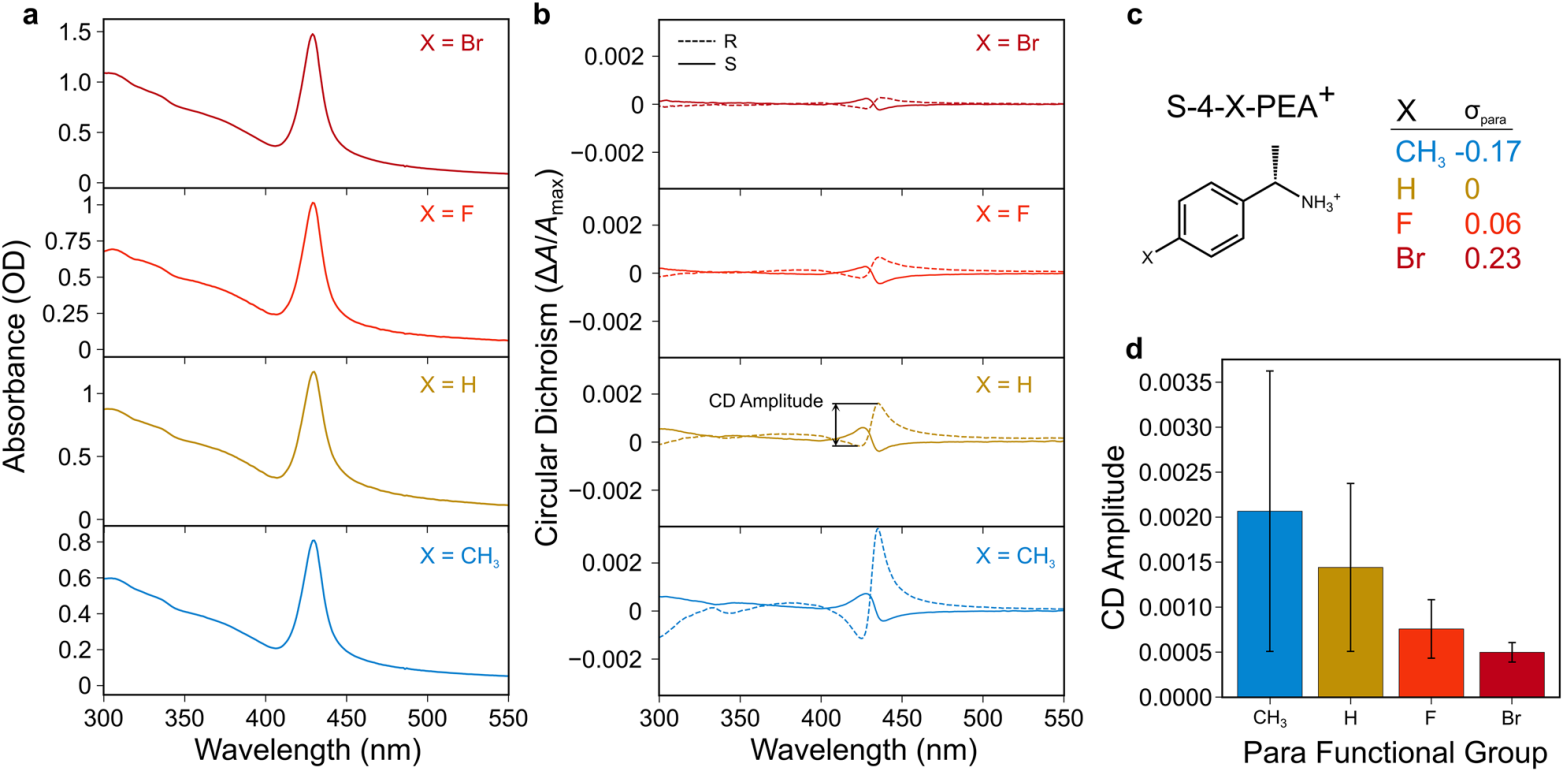
Optical properties of chiral perovskite QDs. UV–Vis absorbance (**a**) and circular dichroism (**b**) spectra of representative samples of CsPbBr_3_ QDs dispersed in toluene following ligand exchange with *S*-4-X-PEABr (solid lines) or *R*-4-X-PEABr (dashed lines), with functional group X = CH_3_, H, F, or Br having *σ*_para_ values in the Hammett equation as indicated in (**c**). Mean CD amplitudes, calculated as the peak-to-trough difference of the bisignate feature associated with the first exciton transition at ~ 429 nm, are shown in the bar plot in panel (**d**), with error bars representing standard deviations among samples from at least 6 different syntheses for each functional group (both *R* and *S* enantiomers are lumped together for this analysis).

After ligand exchange, we observe that the UV–Vis spectra change in minor ways consistent with those of the 2 nm chiral CsPbBr_3_ QDs we have previously investigated (i.e., increase in scattering background, reduced sharpness of optical transitions at energies above the 1st exciton transition, and a modest shift of the 1st exciton peak)^20,29^, from which we infer that the size, shape, and orthorhombic CsPbBr_3_ crystal structure of our QDs are approximately conserved as in these prior reports (we discuss possible implications of size shifts in more detail below in Supplementary Note S1). The CD spectra of the chiral perovskite QDs are dominated by a single bisignate feature occurring in the same spectral region as the lowest-energy exciton peak identifiable in the corresponding UV–Vis spectra (Fig. 2). The existence of this feature shows that chirality is imprinted on the inorganic CsPbBr_3_ QDs, since the ligands lack any transitions in the visible range themselves (Fig. S3). Moreover, comparison of QDs coated with the *R*- and *S*-enantiomers shows spectra with approximate mirror image symmetry. Bisignate features are commonly observed when achiral nanoparticles are capped with a layer of chiral ligands. This spectral signature can be attributed to an energetic splitting of an otherwise degenerate exciton transition for which ground and/or excited states possess different angular momentum; circularly polarized light (CPL) of different polarization excites the transitions with different intensities^33,34^.

We define the “strength” or “amplitude” of these features as the difference between the peak and the trough, when the CD spectrum is expressed as the difference in absorbance Δ*A* between left- and right-handed CPL, normalized by the absorbance of unpolarized light at the exciton peak *A*_max_ to account for variations in solution concentration. We observe that stronger electron-donating functional groups of the ligand produce monotonically increasing average CD amplitude in the 1st exciton peak (Fig. 2d). Here, we quantify electron-withdrawing or -donating character using the Hammett *σ*_para_ parameter, which is defined as the logarithm of the ratio of the ionization equilibrium constant of benzoic acid with the functional group at the para position to that without (i.e., H at the para position)^35^. For the chiral ligands (*R*/*S*)-4-X-PEA^+^ used here, an ethylammonium tethering group takes the place of the carboxyl group in the prototypical case for which *σ*_para_ is defined, and thus the Hammett parameter serves as an empirical metric for how much electron density is withdrawn from the ammonium binding group across the phenyl ring. The CD amplitude has a moderate inverse correlation with *σ*_para_, with Pearson coefficient of $r=- 0.54$; the equivalent Spearman coefficient^36^ indicates a slightly stronger correlation (*r*_Spearman_ = − 0.68) if the condition of a linear relationship is relaxed in favor of a more general monotonic relationship. The trend in CD amplitude with this empirical parameter motivates a more detailed investigation of the possible mechanisms of chirality transfer from the ligand to the QD, as well as a means to tune its strength. Note that we observe a substantial variation in CD amplitude even within samples containing the same functional group; however, the trend of CD amplitude with electron-donating character is generally robust when samples prepared in the same batch are considered or when different enantiomers of the chiral ligand are used (see Fig. S4). Thus, we conclude that a systematic trend exists despite considerable statistical noise arising from batch-to-batch variation in the achiral QD synthesis.

Variations in the CD amplitude with the electron-donating character of the functional group could arise for a number of reasons. We classify these explanations into four categories: (1) the perturbation in charge density of the ligand affects the strength of the ligand-QD bonding, leading to changes in surface density of chiral ligands on the QD surfaces; (2) the electronic coupling between the chiral ligand and QD that gives rise to chiral imprinting is modulated by a change in the charge distribution of the ligand, even as the structure of the QD itself remains unperturbed; (3) large-scale aggregation into chiral superstructures increases differential scattering of right- and left-handed CPL, leading to an apparent CD increase; and (4) structural distortion of the perovskite charge cloud and/or the lattice itself, caused by the interaction forces associated with ligand bonding, produces an inorganic structure that is intrinsically chiral. We exclude category (4) from further experimental study, referring to literature and our own prior studies. While Jana et al.^37^ found that structural chirality transfer is possible (for instance, in chiral 2D perovskites such as *R*-(+)- or *S*-(−)-1-(1-naphthyl)ethylammonium lead bromide), strong induced CD at the lowest-exciton transition is observed even for chiral 2D perovskites where the inorganic lattice assumes an achiral space group (e.g., *R*- or *S*-methylbenzylammonium lead iodide)^34,37^. In previous studies, we examined the role of structural distortions in chiral CsPbBr_3_ QDs, and concluded that, although distortions may give rise to weak, spectrally diffuse CD, they do not account for the much stronger and better-resolved bisignate feature associated with the lowest-exciton transition^29^. Because this bisignate feature is a hallmark of chiral imprinting, is the dominant feature in our CD spectra, and manifests in the absence of a structural distortion, we do not consider this mechanism further in the context of functional group substitution on the chiral ligand. The other categories of ligand-QD interaction are considered in detail below.

### Ligand surface density effects

Before considering specific mechanisms of chiral imprinting, we first explore the possibility that the density of chiral ligands on the QD surfaces is systematically affected by the chemistry of the para position functional group. It is generally agreed that the surfaces of colloidal CsPbBr_3_ nanocrystals tend to be Br^−^-terminated (as illustrated in Fig. S2), and that positively charged ammonium ligands attach at the interstitial spaces between [PbBr_6_] octahedra on the QD surfaces, similar to the bonding scheme in solid-state hybrid organic–inorganic perovskites^32,38^, although this simplified picture may be complicated by the irregular shapes of very small nanocrystals^20,29^. Thus, it is possible that varying the strength of the ammonium-bromide electrostatic interaction may change the chiral ligand surface density. Because the strength of imprinting is expected to scale with chiral ligand surface density (albeit non-linearly)^30^, ligand binding strength could affect the overall CD amplitude. Note, however, that electron-withdrawing functional groups on the phenyl ring are expected to increase the positive charge on the ammonium group, which should increase the electrostatic attraction with the nanocrystal surface, leading to greater densities of chiral ligands and correspondingly stronger imprinting. To approximate the interaction of the ligand with the Br-rich QD surface, we used density functional theory (DFT) to calculate the bond dissociation energy of the (*R/S*)-4-X-PEA^+^-Br^−^ ion pair, and found that it monotonically increases with *σ*_para_ (Table S1), corroborating this intuitive picture. As noted above, we observe the opposite of what we would expect if ligand bonding strength were dominant: the more electron-withdrawing the functional group, the weaker the chiral imprinting. This evidence alone suggests that ligand surface density-related effects do not account for the trend in CD amplitude.

**Figure 3:**
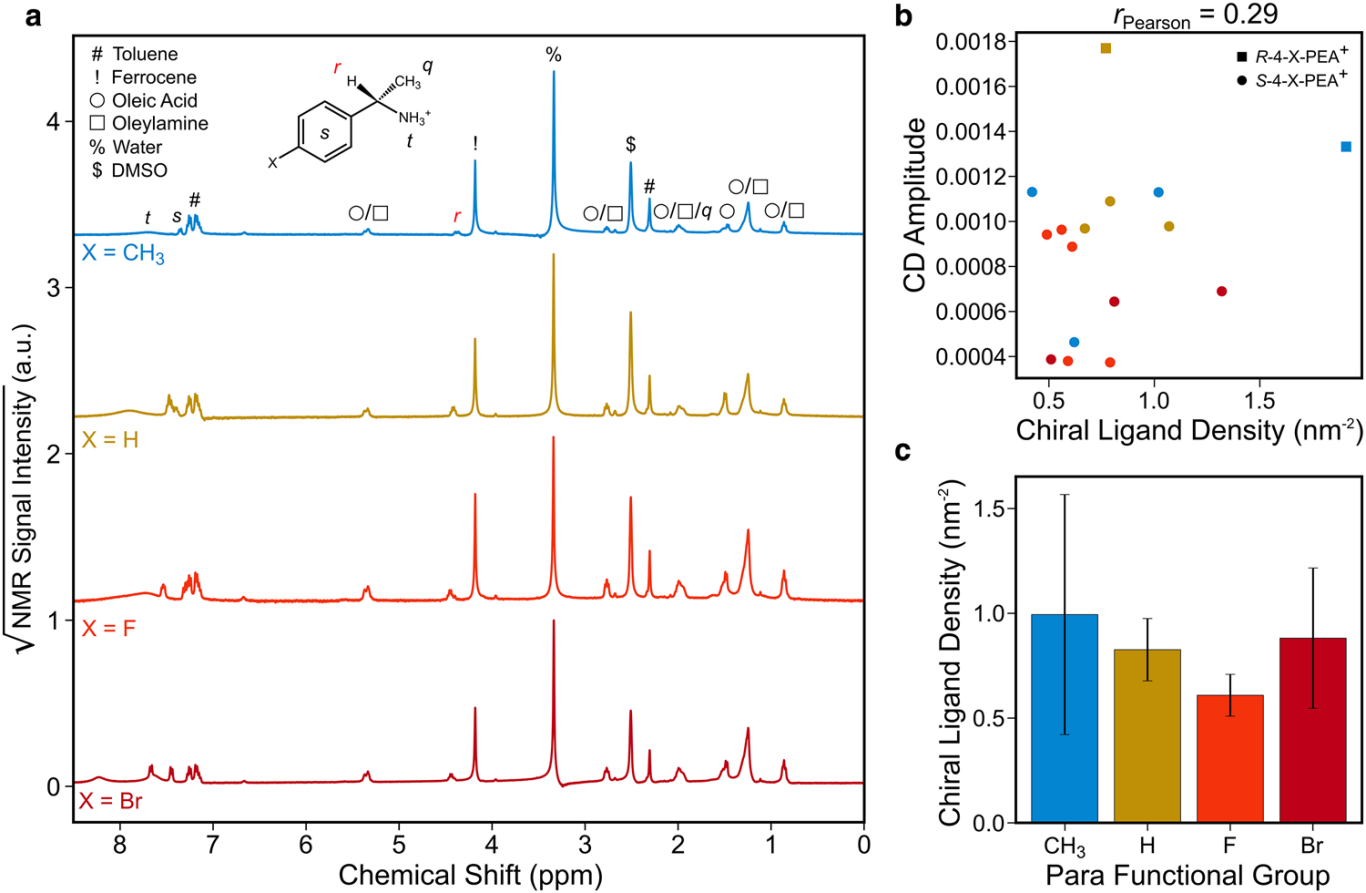
Estimation of chiral ligand density on chiral perovskite QD surfaces by NMR measurements. NMR spectra (**a**) of CsPbBr_3_ QDs decorated with chiral *R/S*-4-X-PEA^+^ ligands digested in DMSO-d_6_, displayed on square root scale to emphasize low-intensity peaks. The peak corresponding to the lone hydrogen atom attached to the chiral center (labeled *r* in red) is used to calculate the surface density of chiral ligands attached to the QD surfaces. Peaks related to oleic acid and oleylamine are visible, which we have previously observed in NMR spectra of achiral CsPbBr_3_ particles^29^, and their presence here indicates that the chiral ligands do not entirely displace the native ones from the QD surfaces. Ferrocene was deliberately added to enable signal intensity calibration, and small amounts of toluene and water persist in the particles even after purification. The correlation (**b**) between CD amplitude and the density of chiral ligands on the QD surfaces estimated from NMR measurements is modest, and there is no appreciable trend of the chiral ligand surface density with electron-withdrawing character of the functional group (**c**). In (**c**), the bars represent the mean values among sample groups, and the error bars represent standard deviations.

To quantify the density of chiral and achiral ligands on the QD surfaces directly, we digested a subset of the *R*- and *S*-4-X-PEA^+^-imprinted QDs in DMSO-d_6_ and performed nuclear magnetic resonance (NMR) measurements on their solutions (with ferrocene added as a standard to calibrate signal intensity). Representative NMR spectra for each ligand are shown in Fig. 3a. We quantify the concentration of *R*/*S*-4-X-PEA^+^ in solutions obtained from a known mass of QDs using the peak corresponding to the hydrogen atom attached to the chiral stereocenter at ~ 4.4 ppm, while the amount of both achiral ligands can be determined using the triplet peak at ~ 0.9 ppm that arises from the terminal methyl group of the alkyl chain in both oleic acid and oleylamine. From these concentrations and the average sizes of the QDs inferred from the exciton peak position in the UV–Vis spectra, we can calculate the surface densities of chiral and achiral ligands (expressed as ligands/nm^2^ of QD surface area). In all cases, the chiral ligands account for a majority of the total number of organic ligands attached to the QD surfaces (Fig. S5), demonstrating the qualitative expected association of the induced CD with their presence. However, plotting the CD amplitudes of the corresponding samples against chiral ligand density shows that there is only a modest correlation between imprinting strength and chiral ligand surface density (Fig. 3b). Although one might expect an increasing chiral ligand density as *σ*_para_ increases, because of the QD-ligand electrostatic attraction, no systematic variation of the chiral ligand density with the electron-withdrawing character of the functional group is evident (Fig. 3c). These results indicate that chiral ligand surface density effects may have a weak influence on a sample-to-sample basis, but they do not adequately explain the observed effect of the functional group on CD intensity.

### Electronic-coupling effects

Having shown that changes in chiral ligand surface density (category 1) are unlikely to account for the variation in chiral imprinting strength induced by ligand functionalization, we now consider possible manifestations of electronic coupling between the ligand and QD, which may include: (i) electrostatic perturbation of the QD exciton states by the static electron density of the ligand^39^; (ii) dipole–dipole coupling between excitonic transitions in the ligand and QD, treated as electronically separate entities^40,41^; and (iii) chiral symmetry transfer via hybridization of the ligand and QD wavefunctions^33^. To interrogate the relevance of each mechanism, we performed DFT calculations of potentially important characteristics of the chiral ligands and probed their correlations with our experimental observations.

In the first mechanism, the electron density of the chiral ligand in its ground state perturbs the QD excitonic states, conferring optical activity onto the transitions between them. For example, Goldsmith et al.^39^ modeled induced CD on a cubic Au nanocluster with adsorbed chiral molecules, employing a “particle-in-a-box” model for the unperturbed states and treating the perturbation from the ligand as a Coulombic potential arising from several nearby point charges in a chiral arrangement. To understand whether changing the functional group significantly affects the effective charge distribution in the vicinity of the QD surface, we calculate the positions of atoms in the groups attached to the ligand’s stereogenic center. We found that changes in these atomic positions are minimal (Fig. S6), and infer that the resulting variations in the strength of the chiral perturbation are unlikely to be substantial.

To investigate the second proposed mechanism, we invoke the coupled oscillator model, which is frequently used to explain the origin of bisignate CD features in molecules with two adjacent chromophores^40^. Dipole–dipole coupling between optical transitions of these two moieties gives rise to a non-vanishing scalar product of electric dipole transition moment (EDTM) ${\varvec{\upmu}}$ and magnetic dipole transition moment $\mathbf{m}$; consequently, the associated transition inherits CD^29,40^. This model assumes that wavefunction overlap between the chromophores is negligible, and their interactions may be described as a purely electrostatic coupling of transition dipole moments (even if the charges involved are not strictly stationary). As applied to the case of chiral QDs, ${\varvec{\upmu}}$ is characteristic of the exciton transition of the unperturbed QD, while $\mathbf{m}$ arises from the helical deflection of this transition’s electron charge displacement that is induced by the interaction with the EDTM of the chiral ligand^40^. If this mechanism dominates the induced CD of the CsPbBr_3_—(*R/S*)-4-X-PEA system, we expect the magnitude and/or orientation of the ligand’s EDTM to change when the functional group is altered, which may then increase or decrease the CD amplitude. A hallmark of the coupled oscillator model is that the apparent Davydov splitting, $2{V}_{12}$, of the bisignate CD feature (estimated as the peak-trough energy difference) should be proportional to its amplitude; here, ${V}_{12}$ is the dipole–dipole coupling energy between the EDTMs of the exciton transition and that of the ligand. We find that the CD amplitude possesses a modest correlation with the apparent Davydov splitting as well as a tendency for the latter to increase with the electron-donating character of the functional group (Fig. S7a,b). This explanation is broadly consistent with a systematic effect of stronger dipole–dipole coupling as the electron-donating character of the functional group increases. As discussed further in Supplementary Note 1, it is most likely that the relative orientation of the ligand and QD EDTMs best accounts for the possible variation in ${V}_{12}$, as neither their magnitudes nor the distance between them should vary appreciably (Figs. S3 and S8). The CD amplitude also possesses a moderately strong inverse correlation (*r* = − 0.53) with the *x*-component of the EDTM of the most significant optical transition between near-frontier orbitals of the ligand (Fig. S7c), although it possesses a much lower correlation with the overall magnitude of its EDTM (*r* = − 0.11). At this stage, we thus consider the coupled oscillator model to be a plausible explanation for the effect of the functional group on the induced CD.

**Figure 4:**
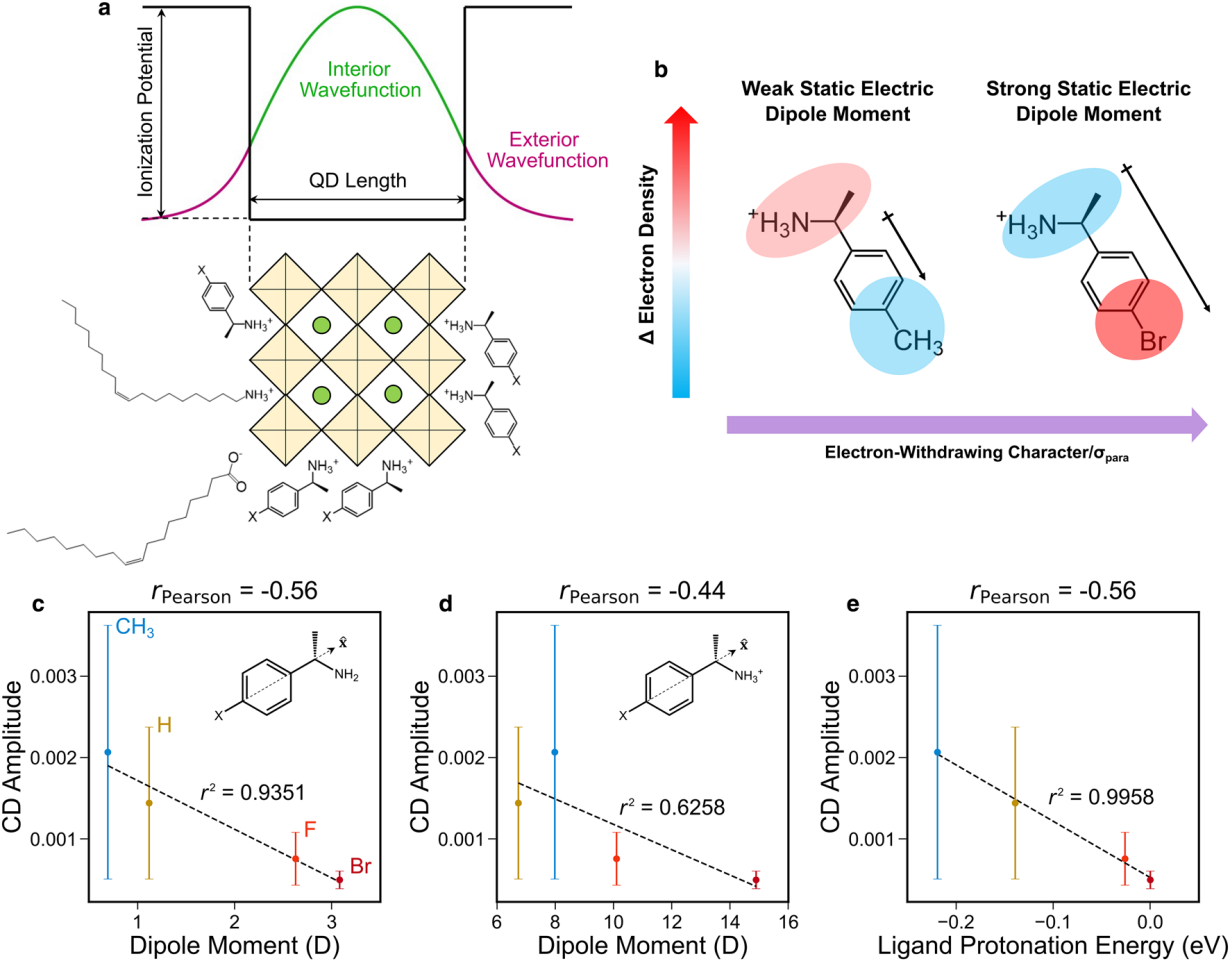
Relationships between observed CD amplitude and overlap of the wavefunctions of the QD exciton and chiral ligand. In strongly confined QDs (such as 2 nm CsPbBr_3_ nanocrystals), there is appreciable tunneling of the exciton wavefunction outside the boundary of the nanocrystal, which may overlap with those of adsorbed ligands (**a**). Changing the para-position functional group modulates the electron density distribution across the ligand (**b**); electron-donating functional groups (e.g., CH_3_) increase electron density nearest the tethering group at which the ligand attaches to the QD surface, thereby enhancing the potential for wavefunction hybridization, while electron-withdrawing groups (e.g., Br) have the opposite effect. Ligand characteristics related to the movement of electron density away from the tethering group—the x-component (along the axis of opposing groups across the phenyl ring) of static electric dipole moment of neutral (*R/S*)-4-X-PEA (**c**) or protonated (*R/S*)-4-X-PEA^+^ (**d**), and the protonation energy of the amine (**e**)—are strongly inversely correlated with the excitonic CD of the corresponding ligand. Note that the Pearson correlation coefficients in (**c**–**e**) refer to the entire dataset, accounting for variation within sample groups, while the *r*^2^ values are calculated with respect to the means of each group (indicated by the circular symbols). Error bars represent the standard deviations of each group. The ligand protonation energy is referenced to the X = Br case.

To evaluate the third hypothesized electronic coupling mechanism—wavefunction hybridization—we consider the extent of overlap between the wavefunctions of the carriers comprising excitons in the QD and the molecular orbitals of the chiral ligand. Ben-Moshe et al.^33^ previously used a QD valence band-ligand HOMO hybridization mechanism to account for the induced CD found in CdSe QDs clad with chiral cysteine ligands, and Jiang et al.^42^ have also proposed this mechanism to operate in chiral perovskite nanoparticles. In this mechanism, hybridization is enhanced by both spatial and energetic overlap of the QD bands with the ligand’s frontier orbitals, and for this reason hybridization is likely to be relevant in strongly confined QD systems where there is a significant probability of carrier tunneling outside the QD boundary. In previous studies of chiral CsPbBr_3_ nanoparticles^29^, we posited that a hybridization mechanism may account for the size dependence of induced CD in these systems, observing an exponential decay of signal amplitude with nanoparticle size. This trend may be attributed to the reduction in tunneling of the exciton wavefunctions beyond the boundary of the confining potential, and subsequent overlap of that density with the adsorbed chiral ligands (as shown schematically in Fig. 4a). Wavefunction coupling may also be modulated through the ligands: here, electron-donating groups should push electron density onto the ammonium tethering group (Fig. 4b), increasing the spatial overlap of the ligand’s molecular orbitals with the QD exciton wavefunction. DFT-calculated values of the static electric dipole moment of the (*R/S*)-4-X-PEA amine, a rough metric of how much charge is drawn across the phenyl ring by the functional group, also have a positive and monotonic trend with the empirical *σ*_para_ parameter (Fig. S9). We find further that induced CD amplitude of the corresponding chiral QDs has a reasonably strong inverse correlation (Pearson correlation coefficient *r* = − 0.56) with the *x*-component of the neutral amine ligand’s static dipole moment (Fig. 4c), and a slightly weaker correlation with that of the conjugate ammonium ligand (Fig. 4d). As a more specific probe of the electron density localized on the ammonium group, we also calculate the protonation energy for the reaction (*R*/*S*)-4-X-PEA + H^+^ → (*R*/*S*)-4-X-PEA^+^. This value correlates almost perfectly with the static dipole moment of (*R*/*S*)-4-X-PEA (Fig. S9), and has a functionally identical correlation with the CD amplitude as the latter (Fig. 4e). Notably, the relationship between the mean CD amplitude for each ligand and the protonation energy is almost perfectly linear. Overall, there are large mutual correlations (*r* > 0.9) among the metrics used to describe the changes in ligand charge density (i.e., *σ*_para_, static dipole moment, and protonation energy), indicating that the empirical *σ*_para_ parameter is a reliable guide to the expected behavior of different ligands (Fig. S9) in spite of the chemical departure from the benzoic acid model system. These relationships are indirect yet compelling evidence that electron-donating groups increase the amplitude of the ligand’s ground state wavefunction near the surface of the QD, where it may overlap with the hole in the perovskite exciton, enhancing chiral imprinting. We therefore conclude that the coupled dipole model discussed previously represents an oversimplified approximation, as it treats the ligand and QD as isolated chromophores and neglects wavefunction hybridization, which appears to provide an important, if not dominant, modality for coupling between QD and ligand.

As noted above, wavefunction hybridization may be influenced by the energetic alignment of the participating states as well as their spatial overlap. To understand the extent to which energetics plays a role, we calculated the relative shifts in the HOMO and LUMO energies of the (*R/S*)-4-X-PEA^+^-Br^−^ ion pairs as an approximation of the ligand in a bound configuration (Fig. S10a). Using an experimentally determined ionization potential of similarly sized CsPbBr_3_ nanocrystals as ~ 5.3 eV^43^ and approximating the band gap using the lowest exciton transition energy of ~ 2.9 eV, we expect Type I alignment between the perovskite bands and ligand frontier orbitals, with the former straddled by the latter. The calculated HOMO and LUMO both shift upwards as *σ*_para_ decreases, and are correlated with CD amplitude at similar levels to those of the electron distribution metrics discussed above (*r* = 0.56 and 0.55, respectively; see Fig. S10b,c). Since the ligand HOMO lies closer to the QD valence band than does the LUMO to the conduction band, we conclude that increased HOMO-VB hybridization with increasing electron-donating character of the functional group may be facilitated by energetic alignment of the participating states as well as their spatial overlap.

### Nanoparticle aggregation effects

Finally, we consider the possibility that the QDs may aggregate into superstructures with length scales up to hundreds of nanometers. Perovskites integrated into such supramolecular structures have been reported to show chiroptical properties, such as circular dichroism and circularly polarized photoluminescence^44^. Similarly, it has been found that, following ligand exchange, chiral perovskite nanoparticles can form large sheet-like structures in which the CD amplitude scales with their lateral size, suggesting that imprinting may be amplified as the dimensions of the nanostructures approach the wavelength of incident light^45^. There remains controversy surrounding whether such effects dominate the excitonic CD, however, Georgieva et al.^19^, for example, found that aggregation did not meaningfully contribute to the CD of perovskite nanoplatelets bound with a mixture of (*R/S*)-phenethylamine and octylamine-based ligands. To understand the degree of aggregation in our samples, we focus on the sub-band gap portion of the UV–Vis absorption spectra in our chiral QDs. In this spectral region, there is a long tail that increases monotonically with decreasing wavelength; see Fig. 2a. This feature may be attributed to scattering by particles smaller than the wavelength of incident light. The effective absorbance is well fit over the 500–800 nm range by a Rayleigh/Tyndall model (*r*^2^ > 0.94 in all cases, *r*^2^ > 0.99 on average), where the scattering intensity is proportional to the inverse fourth power of wavelength:

$${A}_{{\text{scat}},{\text{eff}}}\left(\lambda \right)=-{{\text{log}}}_{10}\left(1-{\left(\frac{{c}_{1}}{\lambda }\right)}^{4}\right)+{A}_{{\text{bkg}}}$$

Here, ${c}_{1}$ is a constant and ${A}_{{\text{bkg}}}$ is a constant baseline value that represents contributions of wavelength-independent Mie scattering by larger particles with sizes on the order of the wavelength of light. In practice, the latter effect is negligible, and ${A}_{{\text{bkg}}}$ appears to be most strongly affected by variation in the instrument baseline (on average, ${A}_{{\text{bkg}}}$ = 0.01 ± 0.01). There is only a weak correlation between CD amplitude and the scattering coefficient ${c}_{1}$, either normalized to the absorbance of the exciton peak or in absolute magnitude (Fig. S11). Although we observe that ligands with the most strongly electron-withdrawing functional groups tend to yield the lowest scattering coefficients, it seems unlikely that aggregation is a dominant contributor to the trends in the CD spectra. Collectively, the modest associations between scattering coefficients and CD amplitudes suggest that nanoparticle aggregation only slightly modulates the strength of chiral imprinting, but the variation in the CD amplitude is better explained by the wavefunction overlap effects described above.

## Summary and conclusion

We studied how differing para functional groups of chiral (*R*/*S*)-phenethylammonium ligands, which coat the surface of colloidal CsPbBr_3_ quantum dots, affect the strength of the QDs’ CD spectra. We find that electron-donating groups produce stronger induced circular dichroism at the QDs’ first excitonic transition. We have analyzed several possible origins for this trend, and find that it is best explained by the increasing overlap of the ligand and QD excitonic wavefunctions with increasing electron-donating character of the ligand’s functional group. This study provides a chemical design rule to optimize chiral imprinting, not only in perovskite quantum dots, but also in other strongly quantum-confined systems (such as 2D hybrid organic–inorganic compounds), and provides support for the hypothesis that exciton CD in perovskites arises largely from the hybridization of the lead halide valence band with the chiral ligand’s HOMO. This understanding motivates the design of new chiral ligands that may further enhance the outstanding chiroptical and optoelectronic properties of perovskites.

## Methods

### Synthesis of (*R/S*)-4-X-PEABr salts

In a typical synthesis, 0.5 mL of (*R/S*)-4-X-phenethylamine (X = CH_3_: Santa Cruz Biotechnology (both enantiomers); X = H: Sigma Aldrich, ChiPros, > 99.0% (both enantiomers); X = F: Sigma Aldrich, ChiPros, > 98.5% (*R*), Thermo Scientific, ChiPros, 99%, enantiomeric excess 99% (*S*); X = Br: Thermo Scientific, ChiPros, 99%, enantiomeric excess 98% (both enantiomers)) and an equimolar amount of hydrobromic acid (Sigma Aldrich, 48 wt% in H_2_O, ACS grade) were added to 5 mL of absolute ethanol (Decon Labs, anhydrous) in a round-bottom flask and stirred at 0 °C for 2 h. The reaction mixture was then placed in a rotary evaporator (Büchi Rotavapor R-3000) and heated at 50–70 °C under vacuum to drive off the ethanol. Generally, the salt did not crystallize directly after removal of the ethanol but rather formed a melt, which could be induced to freeze through careful cooling. After the salt crystallized, it was redissolved in hot chlorobenzene (Thermo Scientific, 99%) and left to recrystallize overnight. The recrystallized product was washed copiously with toluene (Fisher Scientific, ACS grade) and dried under vacuum for several hours, then stored in an argon-filled glovebox for further use.

### Synthesis and purification of CsPbBr_3_ quantum dots

Perovskite QDs in this work were fabricated via a conventional hot-injection method^20,29,32^. In a typical synthesis, a Cs oleate solution was prepared by adding 200 mg of Cs_2_CO_3_ (Sigma Aldrich, 99.9%) and 0.7 mL of oleic acid (Fisher Scientific, 90%) to 10 mL of 1-octadecene (ODE; Sigma Aldrich, 90%) in a three-neck round-bottom flask (RBF). In a separate three-neck RBF, 320 mg PbBr_2_ (Sigma Aldrich, 99.999%), 2.4 g ZnBr_2_ (Sigma Aldrich, 99.999%), 8 mL oleylamine (Sigma Aldrich), and 8 mL oleic acid were mixed in 20 mL ODE. Both flasks were heated to 130 °C and held at that temperature for 1 h while purging with argon. Afterward, the flasks were sealed and the temperature was raised to 140 °C and held there for a further 45 min to facilitate dissolution of the precursors (note that ZnBr_2_ is used in such large excess that it does not completely dissolve). The Cs oleate and PbBr_2_ flasks were then cooled to 120 °C and 90 °C respectively. Once the temperature stabilized, 2.4 mL of Cs oleate solution was injected into the PbBr_2_ flask, and allowed to react for 3 min, after which the reaction was quenched by immersing the flask in an ice bath. The crude reaction mixture was then collected and stored under argon for approximately 24 h. Excess solids were removed by diluting the reaction mixture in toluene (Sigma Aldrich, anhydrous grade; > 2 parts toluene to 1 part r.m. by volume) and centrifuging it at 6500 rpm at 15 °C for 15 min, affording a clear yellow solution. The solution was then stored overnight to allow the particles to grow to 2 nm; tert-butanol (Fisher Scientific, certified grade) was added at ~ 0.5% by volume to suppress the formation of other, undesired nanostructures. After resting the particles overnight, the solution was centrifuged again at 6500 rpm at 15 °C for 15 min to remove any unwanted aggregates. The 2 nm CsPbBr_3_ QDs were then extracted from the solution by centrifuging it at 10,500–12,000 rpm at − 5 to − 3 °C for 1 h and recovering the pellet; sometimes, this process was repeated several times to accumulate enough pellet material. The pellets were then redispersed in 90 mL of anhydrous toluene and purified by centrifuging again at 12,000 rpm at − 5 °C for 1 h. The pellets were again recovered and redispersed in toluene, and the resulting solutions diluted such that the absorbance of the 1^st^ exciton peak at 428 nm was ~ 2 OD. Note that during the purification process, precautions were taken to avoid exposing the CsPbBr_3_ QDs to air, and most of the solution preparation steps were performed in an argon-filled glovebox. Only at the end of each centrifuge step, when the tubes had to be opened to decant the supernatant, were the CsPbBr_3_ QDs exposed to air for no more than 3–5 min at a time before being returned to the glovebox for further manipulation or ligand exchange.

### Chiral ligand exchange

0.92 M stock solutions of the chiral ligand salts (*R*/*S*)-4-X-PEABr in tert-butanol (Fisher Scientific, certified grade) were prepared in advance, and added to the 2 OD CsPbBr_3_ QD solutions prepared as detailed above such that the final concentration of ligand was 1.84 mM. In all but one case, oleylamine was also added to the solutions at 5% of the concentration of (*R/S*)-4-X-PEABr to help stabilize the 2 nm QD populations and prevent the growth of larger particles. The solutions were incubated at room temperature under argon in the dark for 2 h, then centrifuged at 7500 rpm at 10 °C for 15 min. The pellets were recovered and redispersed in anhydrous toluene, and the resulting solutions were used for optical measurements.

### Optical spectroscopy measurements

UV–Vis spectra were collected using an Agilent 8453 spectrometer, with samples contained in 1 cm × 1 cm quartz cuvettes. Each spectrum was taken with reference to a blank solvent spectrum. Circular dichroism spectra were collected on the same samples using a Jasco J-810 spectropolarimeter with a scan speed of 100 nm/min and bandwidth of 1 nm; the spectra were generally strong enough that signal averaging was unnecessary. As in the UV–vis measurements, a blank toluene baseline spectrum was subtracted from the CD spectra before analysis.

### NMR measurements and calculation of ligand surface density

To prepare samples for ^1^H-NMR measurements, pellets obtained from purification of the chiral particles were dried under vacuum for 1–2 h, then weighed and digested in deuterated dimethyl sulfoxide (DMSO-d_6_; Cambridge Isotope Laboratories, 99.9% D). A 0.02 M stock solution of ferrocene (Thermo Scientific, 99%) in DMSO-d_6_ was added to the digested pellet solution at 0.004 M, and was used to calibrate the intensity of the NMR spectra. NMR measurements were then collected using a Bruker Advance 400 MHz spectrometer. From the ferrocene-calibrated spectra, the total mass of ligands in solution could be calculated, and subtracted from the net pellet mass to yield the mass of CsPbBr_3_. Assuming the particles to possess a cubical shape, the total number of QDs in the sample and hence total surface area could be calculated from the known volume and mass of the unit cell of orthorhombic CsPbBr_3_. Combining the mass of ligand determined from the NMR measurements with this surface area allows the density of both chiral and achiral ligands to be estimated.

### DFT calculations

The structures of the amine and protonated amine are optimized using B3LYP density functionals with the Def2-SVP basis set as implemented in Gaussian 16^46^. The empirical correction to the dispersion energy is also considered using the Grimme’s dispersion with Becke–Johnson damping^47^. The reported energetic and dipole moments are further characterized using the Def2-TZVPP basis with the same the B3LYP functional.

### Supplementary Information

Supplementary Information.

## Data Availability

The datasets generated during the current study are available from the corresponding author on reasonable request.
