# Supplementary material for: Electron-donating functional groups strengthen ligand-induced chiral imprinting on CsPbBr3 quantum dots
Source: Sci Rep. 2024 Jan 3;14:336. doi: 10.1038/s41598-023-50595-2 (PMC10764765; doi:10.1038/s41598-023-50595-2)
Supplement: Supplementary file 1 — Supplementary Information. [file 41598_2023_50595_MOESM1_ESM.docx]

Supporting Information for

**Electron-Donating Functional Groups Strengthen Ligand-Induced Chiral Imprinting on CsPbBr_3_ Quantum Dots**

**Wiley A. Dunlap-Shohl,**^1^ **Nazifa Tabassum,**^1^ **Peng Zhang,**^2^ **Elizabeth Shiby,**^1^ **David N. Beratan,**^2^ **David H. Waldeck**^1,*^

^1^University of Pittsburgh, Department of Chemistry, Pittsburgh, 15213, United States of America

^2^Duke University, Department of Chemistry, Durham, 27708, United States of America

*dave@pitt.edu


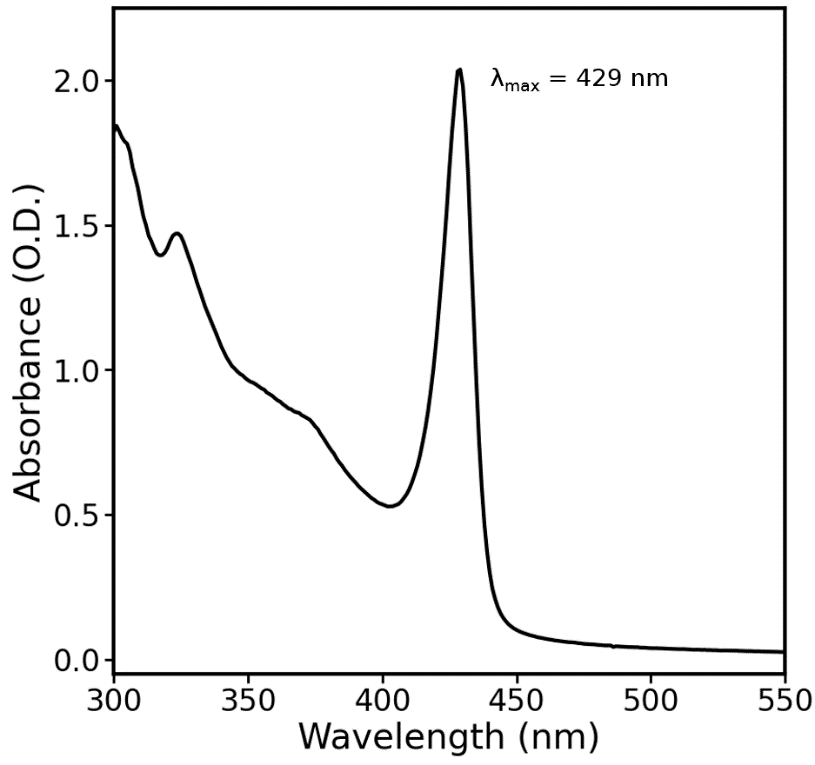


**Figure S1.** Representative UV-vis absorption spectrum of achiral CsPbBr_3_ nanoparticles dispersed in toluene immediately prior to chiral ligand exchange.


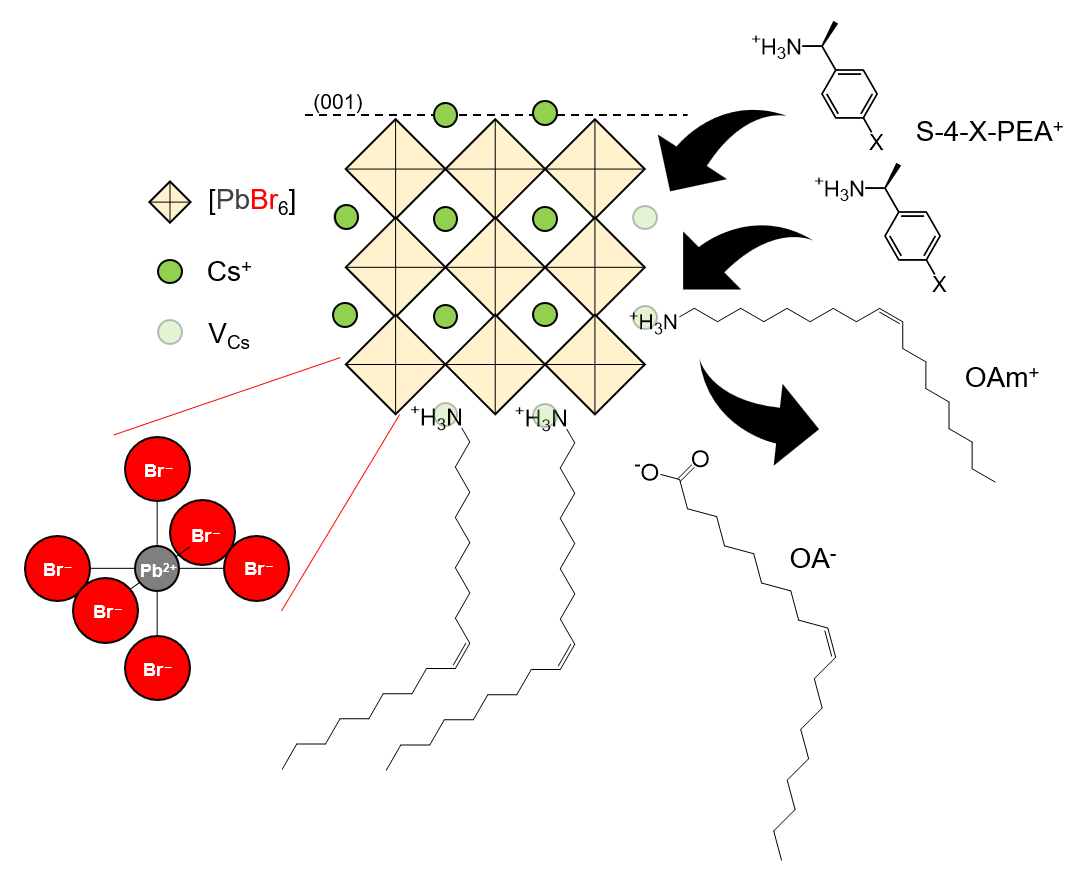


**Figure S2.** Simplified schematic of ligand bonding and exchange in CsPbBr_3_ quantum dots, following Ravi et al.^1^ In this idealized model, the nanocrystals are cubic and terminated on the faces corresponding to the (001) planes of the cubic CsPbBr_3_ crystal structure (or the analogous (001) and (110) planes in the orthorhombic structure). In this conformation, the nanocrystal surfaces are terminated by the negatively charged faces of unbroken [PbBr_6_] octahedra and the Cs^+^ cations residing in the interstitial spaces between them (as they would if the lattice were continued indefinitely, as in a perfect crystal). During synthesis, these Cs-sites may be easily filled by the polar head groups of oleylammonium (OAm^+^) ligands formed by proton exchange between oleic acid and oleylamine reagents. The conjugate oleate (OA^-^) ligands are also present, but are not believed to be directly bound to the surfaces in strictly cubic nanocrystals. During ligand exchange, chiral ligands (e.g., S-4-X-PEA^+^) may easily substitute for the native OAm^+^ ligands without significant surface restructuring since their ammonium tethering groups enable them to bind at the same sites. We emphasize, however, that this scheme is an idealized picture most appropriate for larger nanocrystals that clearly possess a cubic shape. Previous work indicates that ~2 nm CsPbBr_3_ quantum dots may deviate significantly from a cubic shape,^2,3^ implying that other ligand bonding modes may become important (e.g., Pb-oleate bonding may become favorable if surfaces include partial [PbBr_6_] octahedra).


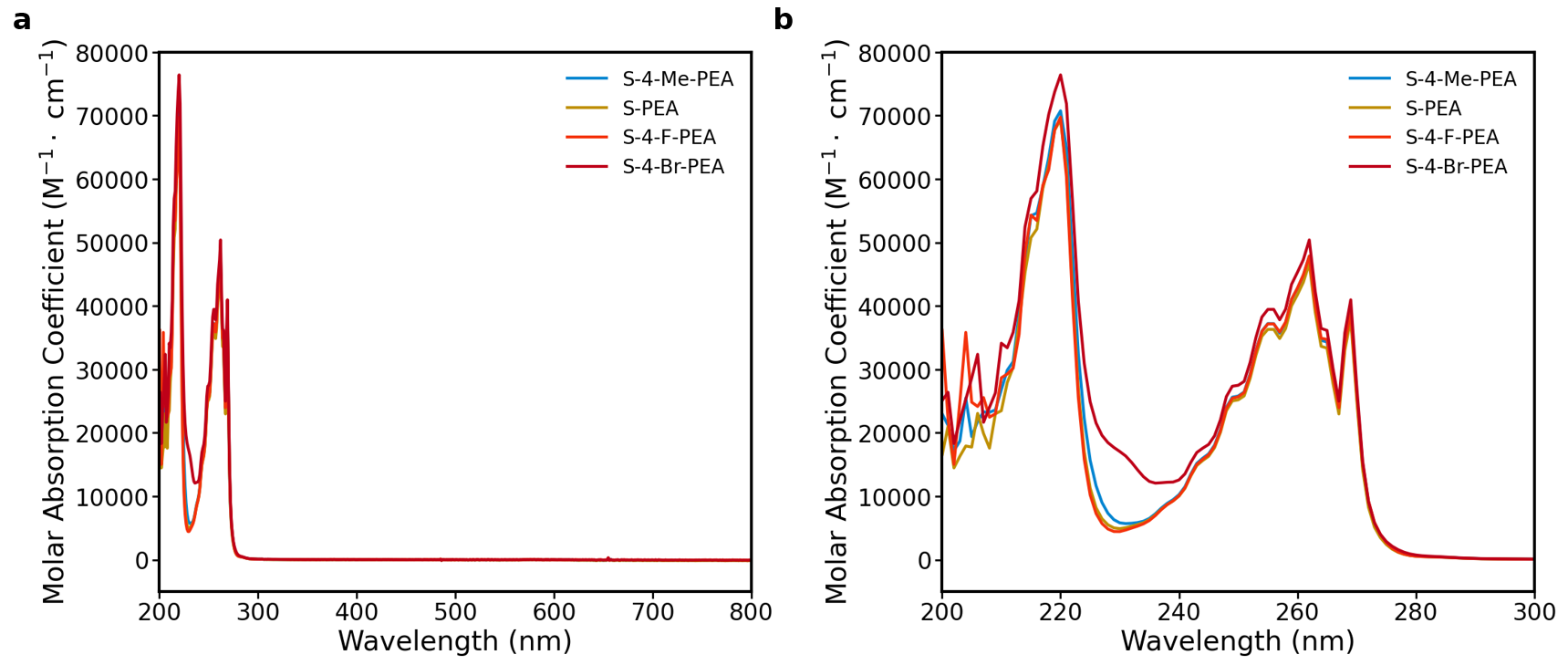


**Figure S3.** Molar absorption coefficients of 25 μM solutions of unprotonated S-4-X-PEA amines in methylcyclohexane calculated from UV-vis absorption measurements shown over the UV and visible range (a) and focused on the ligand absorption features in the UV (b).


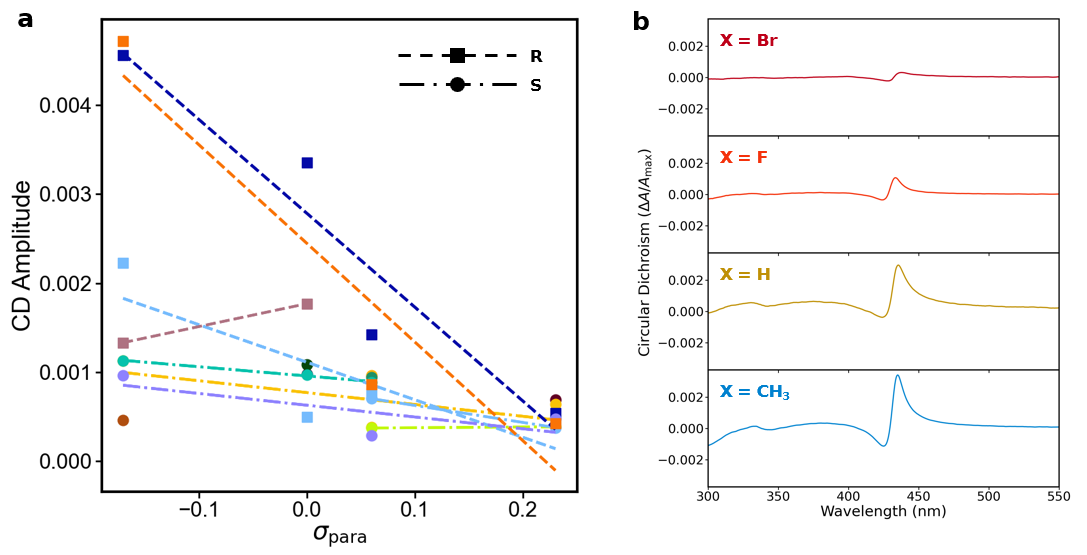


**Figure S4.** (a) CD amplitudes of the data used to construct Figure 1d, plotted against the Hammett *σ*_para_ parameter. Data from different batches are plotted as circles in different colors; trend lines for batches with multiple samples are shown as dashed lines. In most cases, the slope of the trend line is negative, showing that samples within a given batch tend to be representative of the trends observed when the data are taken in aggregate. (b) CD spectra of the samples indicated by the dark blue symbols in panel (a), illustrating the trend for a batch containing all four types of ligand.

**Table S1.** DFT-calculated bond energies of (*R/S*)-4-X-PEA+-Br- complexes. Here, larger bond energies represent more tightly bound systems.

| Functional group X | σ_para_ | Bond energy (eV) |
| --- | --- | --- |
| CH_3_ | -0.17 | 5.03 |
| H | 0 | 5.10 |
| F | 0.06 | 5.19 |
| Br | 0.23 | 5.20 |


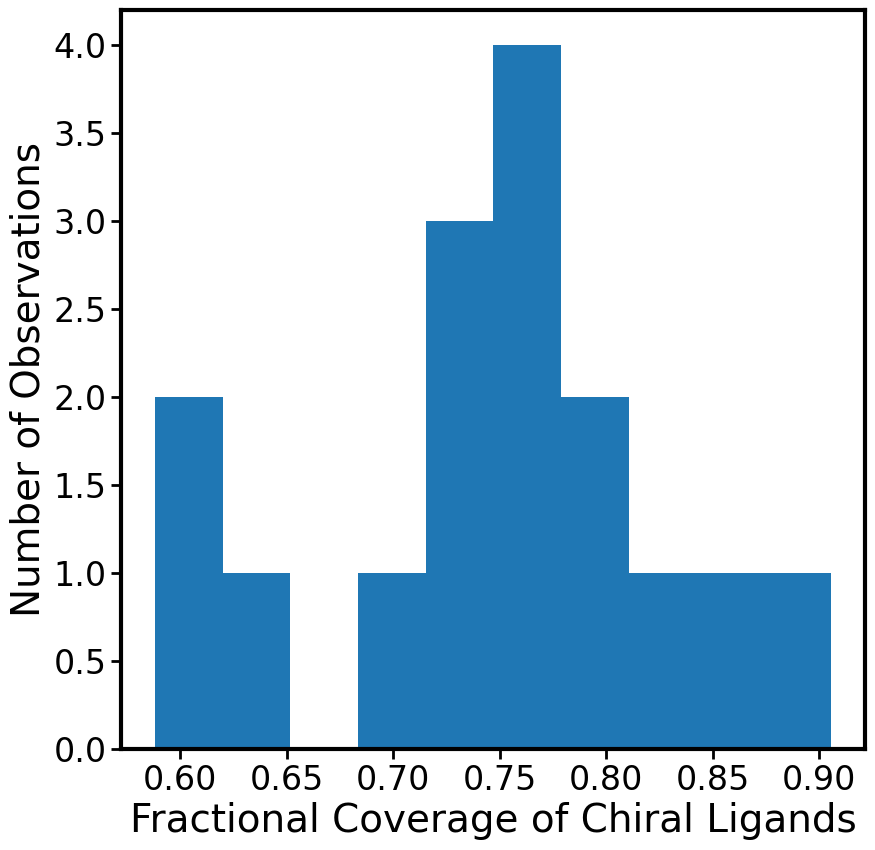


**Figure S5.** Histogram of the fraction of chiral ligands adsorbed on the surfaces of perovskite quantum dots, as determined by NMR measurements – that is, $f_{\mathrm{chiral}}=S_{\mathrm{chiral}}/(S_{\mathrm{chiral}}+S_{\mathrm{achiral}})$, where $S_{\mathrm{chiral}}$ and $S_{\mathrm{achiral}}$ are the respective surface densities of chiral and achiral ligands. In every case, chiral ligands account for the majority. Each observation in the histogram corresponds to a single data point in Figure 3b.


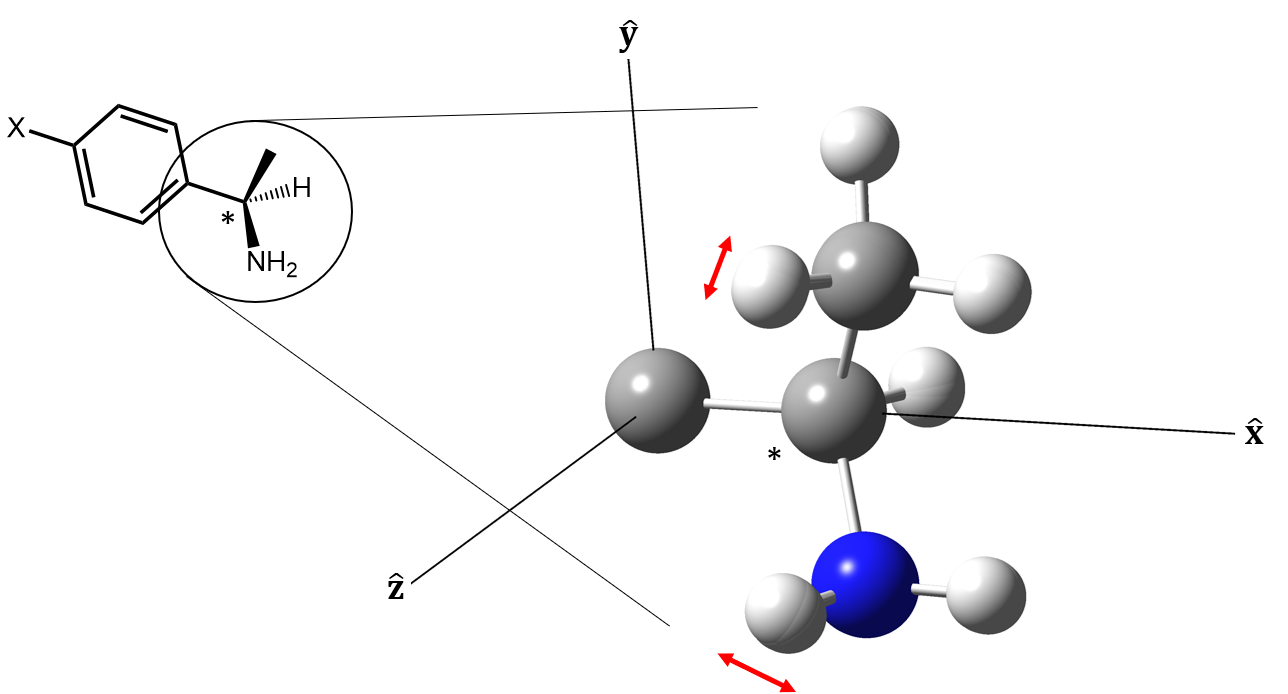


**Figure S6.** DFT-calculated positions of atoms attached to the stereocenter of *R*-4-X-PEA (denoted by an asterisk) for different functional groups X = CH_3_, H, F, and Br. All four structures are superimposed on one another to emphasize that shifts in these positions are minimal; the most significant are indicated by red arrows.


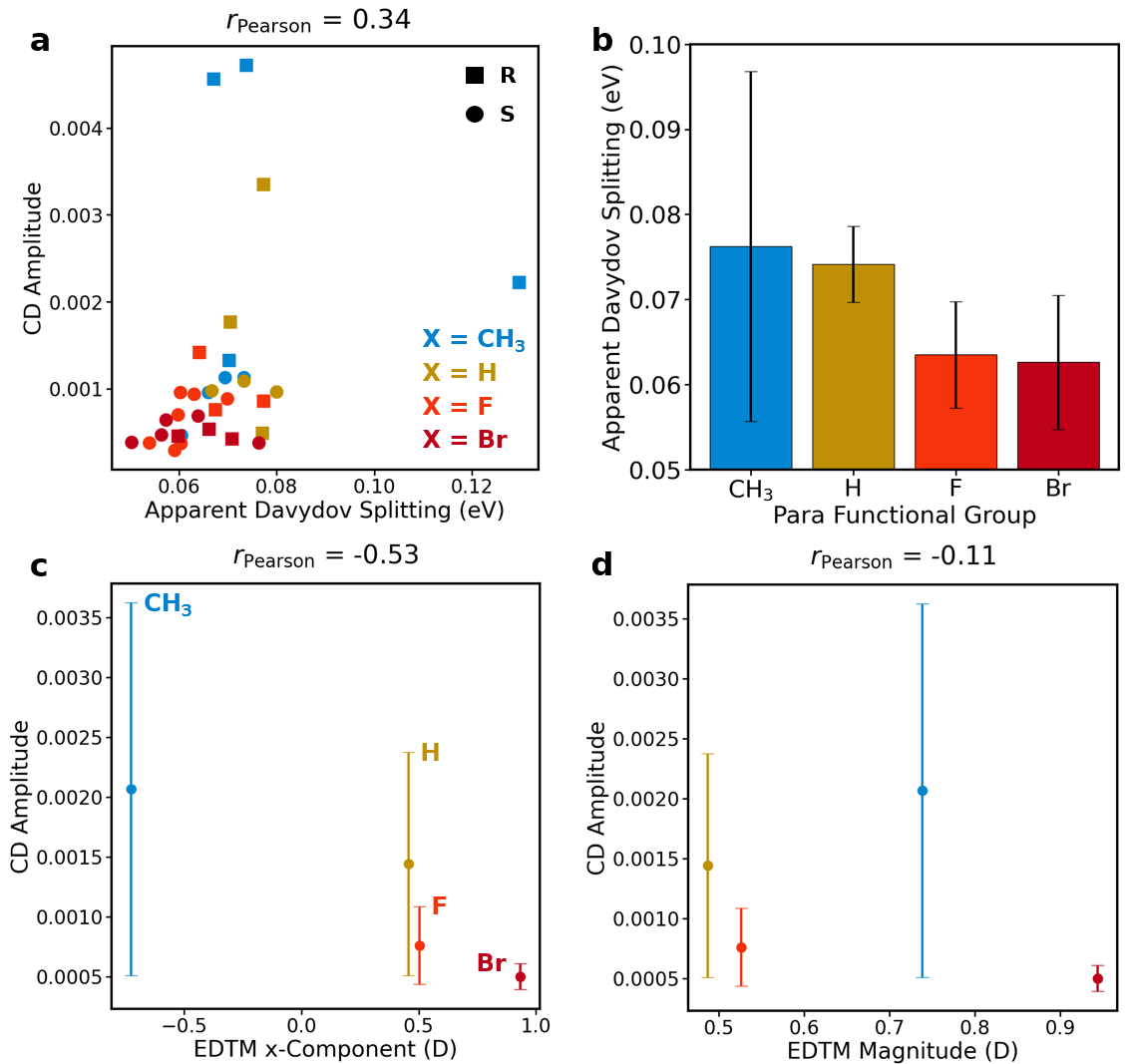


**Figure S7.** Variation of excitonic CD amplitude with apparent Davydov splitting (a); apparent Davydov splitting broken down by chiral ligands with different functional groups (b); CD amplitude versus x-component (c) and magnitude (d) of the most significant electric dipole transition moment between near-frontier orbitals of the chiral ligands.


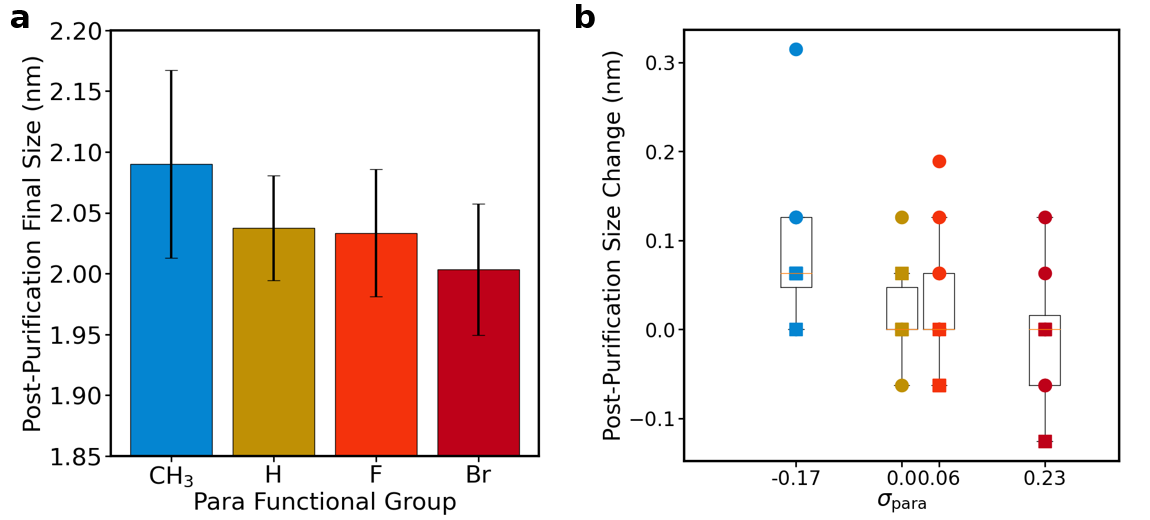


**Figure S8.** Quantum dot sizes after ligand exchange (b) and shifts relative to their sizes before ligand exchange (c), showing that the more electron-donating the functional group, the larger the particles tend to grow (while they may even shrink in cases where the functional group is neutral or electron-withdrawing).

**Supplementary Note 1.** To better understand potential origins of the CD trend under the coupled oscillator model, we note that $V_{12}=\frac{\mu_{1}\mu_{2}}{r_{12}^{3}}(\mathbf{e}_{1}\cdot\mathbf{e}_{2}-3(\mathbf{e}_{1}\cdot\mathbf{e}_{12})(\mathbf{e}_{2}\cdot\mathbf{e}_{12}))$ may be broken down into three parts: (a) a contribution from the magnitude of the ligand EDTM $\mu_{2}$ (we assume the NP exciton EDTM $\mu_{1}$ remains constant); (b) the effective distance between the ligand and the exciton in the nanoparticle $r_{12};$ and (c) a geometric factor representing the relative orientations of the ligand and nanoparticle exciton EDTMs. These orientations are represented by the unit vectors **e**_1_, **e**_2_, and **e**_12_, which point in the directions of **μ**_1_, **μ**_2_, and **r**_12_ (the displacement vector from nanoparticle to ligand dipole positions), respectively. We can rule out the significance of (a) by noting that the molar absorption coefficient of the S-4-X-PEA amines, which is proportional to the square of the EDTM, barely changes as the functional group is varied (**Figure S2**). We can challenge (b) by noting that the particles do show a slight systematic difference in size as a result of ligand exchange (**Figure S8**), as estimated from the exciton peak shifts of the chiral QDs relative to the achiral ones: for X = CH_3_, H, F, and Br, Δλ_max_ = 1.5 ± 1.5 nm, 0.3 ± 0.9 nm, 0.5 ± 1.2 nm, and -0.1 ± 1.2 nm, respectively (expressed as average ± standard deviation). Electron-donating functional groups tend to result in larger particles, although the change in size is in most cases not much more than 1 Å, or about 5% of the nominal 2 nm diameter of the particles. Also, the trend in size we observe is opposite to what we would expect if size changes were a dominant effect – i.e., the CD amplitude and Davydov splitting should decrease as the particles become larger, but we instead observe the reverse. By process of elimination, we conclude that should the variation of the Davydov splitting correctly explain the associated trend in CD amplitude, it arises from changes in the ligands’ functional group orientation on the nanoparticle surfaces.


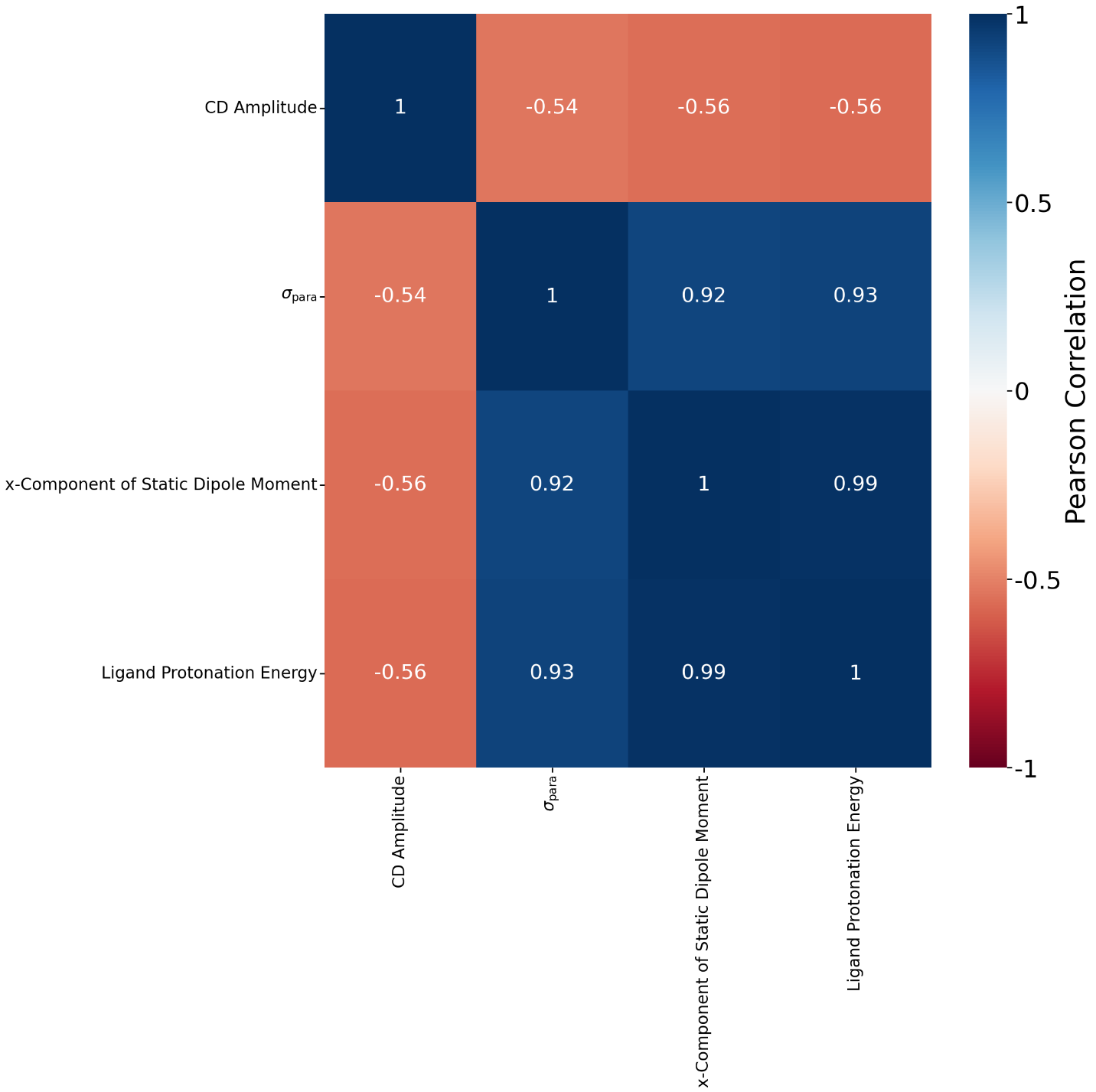


**Figure S9.** Heat map of Pearson correlations between CD amplitude of chiral QDs, Hammett σ_para_ parameter values for the functional groups across (*R/S*)-4-X-PEA ligands, ligand static dipole moment strength, and ligand protonation energy.


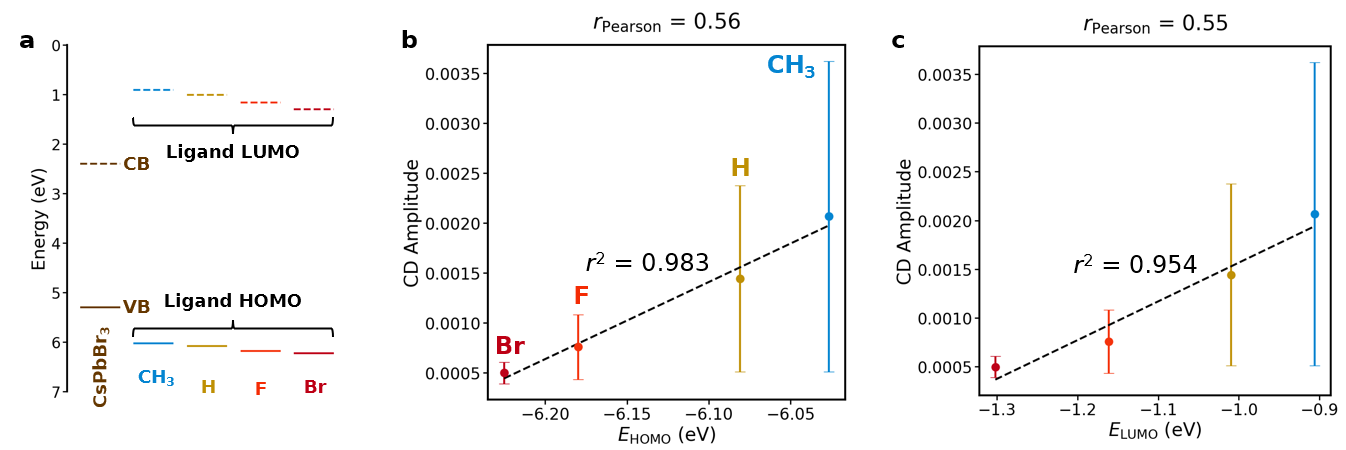


**Figure S10.** Variation of energy level alignment between CsPbBr_3_ QDs and (*R/S*)-4-X-PEA^+^-Br^-^ ion pair complexes(a), showing Type I alignment between QDs and ligands. Variation of chiral QDs’ CD amplitude with the DFT-calculated HOMO (b) and LUMO energy (c) of the above complexes as a result of changing the functional group. Pearson correlation coefficients characterize the entire data set; *r*^2^ coefficients characterize the linear trend in mean CD amplitudes for each sample group (circular symbols).


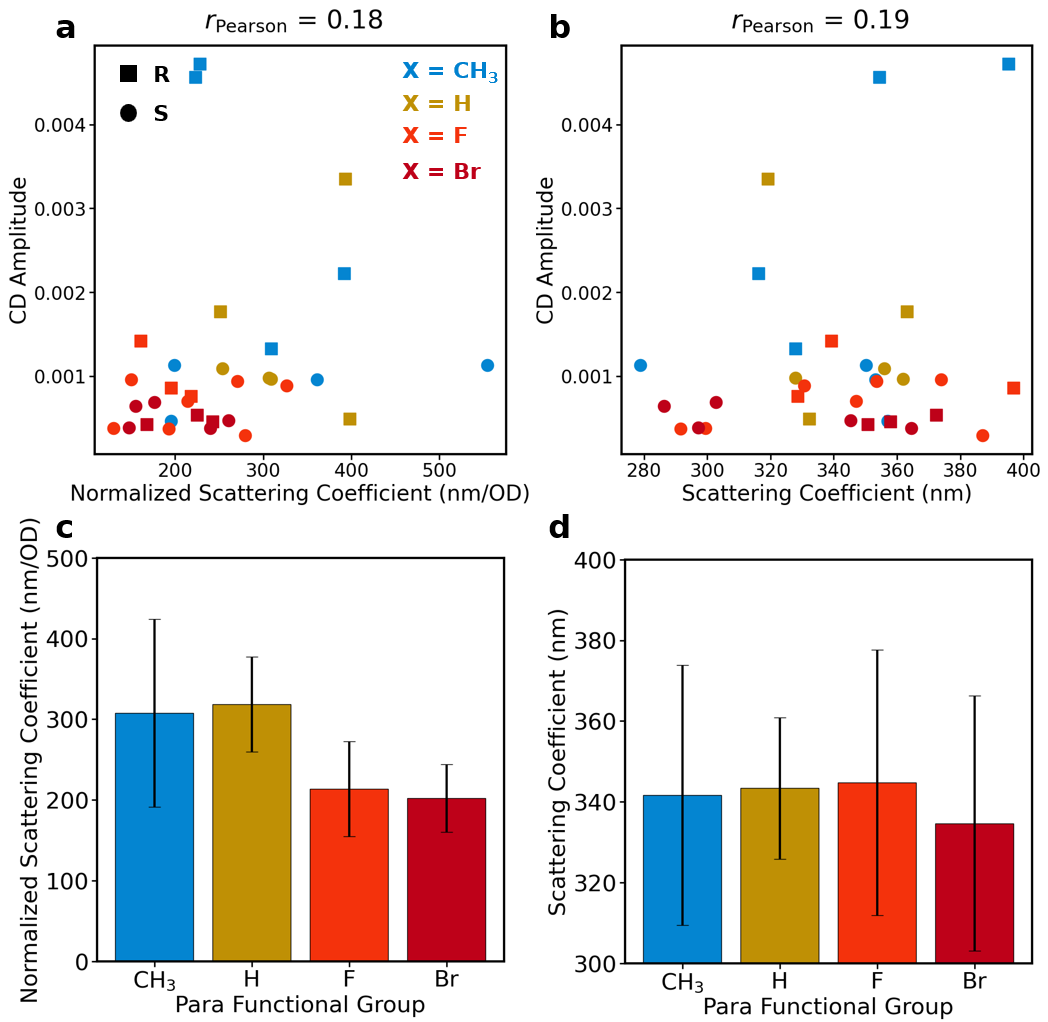


**Figure S11.** There exists only a relatively weak correlation between CD amplitude and the scattering coefficient in a Rayleigh/Tyndall-like model, either normalized by the absorbance of the 1^st^ exciton peak (a) or taken as its raw value (b); the trend in the normalized scattering coefficient with electron-withdrawing character of the para functional group (c) slightly resembles that of the CD amplitude in that samples with electron-donating groups tend to have slightly stronger scattering, but there is no evident trend for the raw scattering coefficient (d).

**References**

1. Ravi, V. K. *et al.* Origin of the Substitution Mechanism for the Binding of Organic Ligands on the Surface of CsPbBr _3_ Perovskite Nanocubes. *J. Phys. Chem. Lett.* **8**, 4988–4994 (2017).

2. Debnath, G. H., Georgieva, Z. N., Bloom, B. P., Tan, S. & Waldeck, D. H. Using post-synthetic ligand modification to imprint chirality onto the electronic states of cesium lead bromide (CsPbBr_3_) perovskite nanoparticles. *Nanoscale* **13**, 15248–15256 (2021).

3. Tabassum, N., Georgieva, Z. N., Debnath, G. H. & Waldeck, D. H. Size-dependent chiro-optical properties of CsPbBr_3_ nanoparticles. *Nanoscale* **15**, 2143–2151 (2023).
